# Prevalence and Factors Associated with Depression among HIV/AIDS-Infected Patients Attending ART Clinic at Jimma University Medical Center, Jimma, Southwest Ethiopia

**DOI:** 10.1155/2020/5414072

**Published:** 2020-08-05

**Authors:** Beyene Dorsisa, Gutema Ahimed, Susan Anand, Tariku Bekela

**Affiliations:** School of Nursing and Midwifery, Jimma University, Ethiopia

## Abstract

**Background:**

HIV is a chronic life-threatening illness and, like other similar chronic and stigmatizing illnesses, can be stressful to manage. Depression is a common mental health problem that deteriorates the quality of life of people with HIV/AIDS and found to be a strong predictor for noncompliance to antiretroviral therapy treatment. Therefore, epidemiological evidence on the factors associated with depression among patients with HIV/AIDS can contribute towards effective and efficient preventive health care strategies for this population.

**Objectives:**

To assess the prevalence and factors associated with depression among HIV/AIDS-infected patients attending ART clinic at Jimma University Medical Center, Jimma, Southwest Ethiopia, in 2018.

**Methods:**

This study followed an institution-based cross-sectional quantitative study design. A simple random sampling method yielded 303 participants who were interviewed from April to May 2018, using a pretested questionnaire, followed by their card review. The SPSS version 23 was used for bivariate analysis which was used to find out the significance of association. Variables that showed association in bivariate analysis at *p* value < 0.25 were entered to multivariable logistic regressions to control for confounders, and the significance of association was determined by 95% confidence interval and *p* value < 0.05.

**Results:**

The point prevalence of depression was 94 (31%). Variables like sex (AOR = 0.510 (95%CI = 0.264‐0.986)), marital status (AOR = 3.610 (95%CI = 1.649‐7.901)), opportunistic infection (AOR = 3.122 (95%CI = 1.700‐5.733)), and medication adherence (AOR = 0.470 (95%CI = 0.266‐0.831)) were significantly associated with depression. *Conclusion and Recommendation*. From the findings of this study, it is possible to conclude that depression was highly prevalent among people living with HIV/ADS. Sex, marital status, opportunistic infection, and medication adherence were found to be associated with depression and need attention from the health professional working in the ART clinic.

## 1. Introduction

Depression is one of the major mental health problems categorized under mood disorders and characterized by persistent experience of a depressed mood and loss of interest [1] and accompanied by symptoms like change in appetite, disrupted sleep patterns, increased or diminished activity level, impaired attention and concentration, and markedly decreased feelings of self-worth.

In severe forms, death wish or attempts to take one's life are present. Early diagnosis is essential, as in addition to the mental suffering, it causes significant social and occupational impairment [2]. HIV/AIDS is one of the global burden diseases, recognized as a serious public health problem and has spread throughout the world affecting all population [3].

It is a chronic and life-threatening illness and overwhelming to the individual [4]. Its life-threatening nature may induce fear of impending mortality. The medical squeal of HIV infection its associated opportunistic infections and side effects of antiretroviral treatment can mimic symptoms of depression (fatigue, concentration problems, somatic symptoms, decreased appetite, and weight loss [5]). When patients with HIV/AIDS develop depression, it accentuates the disease burden and is associated with poor health outcomes. It is also linked with poor antiretroviral therapy (ART) adherence resulting in loss of therapeutic effect, besides having a negative impact on the quality of life of people with HIV/AIDS. Unfortunately, depression is not diagnosed in majority of patients, and therefore, the condition goes untreated [6].

Reasons for high prevalence of depression among people living with HIV/AIDS (PLWHA) could be many [7]. Studies have revealed that depression is associated with different sociodemographic and health-related factors. It is persistently highly prevalent among the poor [8, 9]. Studies in Botswana and Uganda, for example, revealed that depression was associated with food insecurity and the sex of the persons infected with HIV/AIDS [8–10]. The study carried out in Tigray, North Ethiopia, revealed that it was associated with the place of residence, economic status, and occupational status [11]. Depression tends to weaken a person's capacity for judgment, thereby predisposing for risky behaviors [12]. Recent data on the topic is lacking in Jimma; therefore, this study is aimed at determining the prevalence of depression and associated factors among PLWHA who are in ART follow-up at JUMC, Jimma.

Research evidence on the magnitude of depression among PLWHA is important to design more effective treatment programs for the management and prevention of its consequences and to improve their quality of life.

This study will provide research evidence for stakeholders on the magnitude of depression among PLWHA, in order to implement clinical and community-based intervention programs for the management and prevention of depression among PLWHA, aimed at improving their quality of life.

## 2. Methods and Materials

### 2.1. Study Area and Period

Jimma University Medical Center is found in Oromia region, southwest of Ethiopia in Jimma town, which is located 346 km to the southwest of Addis Ababa and provides health services for population in the town and the surrounding districts. In the town, there are different health facilities: four health centers, one general hospital and one specialized hospital currently, and one health center, and the two hospitals are giving ART service for people living with HIV/AIDS. In Jimma University Medical Center Hospital, there is a separate ART clinic at which care and follow-up are given for 3075 PLWHA. The study was conducted from April 3 till May 2018.

### 2.2. Study Design

Institutional-based cross-sectional study design was used.

### 2.3. Source Population

The source population were all PLWHA attending ART Clinic at Jimma University Medical Center.

### 2.4. Study Population

The study population were all PLWHA attending ART clinic at JUMC and who were included in to the sample

### 2.5. Eligibility Criteria

#### 2.5.1. Inclusion Criteria

- Participants aged older than or equal to 18years
- Participants who are on routine appointment receiving ART treatment

#### 2.5.2. Exclusion Criteria

- Bereavement within 3 months
- Female patient in postpartum period
- Those who have not done their CD4 count in six months preceding data collection

### 2.6. Sample Size Determination

The sample size was determined by using single population proportion formula. The following assumptions were made: marginal error (*d*) that was tolerated in either sides of the true proportion to be 5%, using 95% confidence level,*α* = 0.05, and the proportion of depression in HIV/AIDS patient taken from a research done in Fitche Zonal Hospital in 2017(*P*) = 76.7%. Those on steroid treatment [8]

(1) $$\begin{array}{c} n=\frac{{\left(Z\left(\alpha /2\right)\right)}^{2}P\left(1-P\right)}{d^{2}},\\ n=\frac{{\left(1.96\right)}^{2}.767\left(1-.767\right)}{{\left(.05\right)}^{2}}=275, \end{array}$$

where *n* is the required sample size/minimum sample size required for the study.

*Z* is the standard normal distribution (*Z* = 1.96) with confidence interval of 95%, and *α* = 0.05.

*P* is the prevalence/population proportion (*P* = 0.767).

*d* is the desired absolute precision, a tolerable margin of error (*d* = 0.05) formula with 10% nonresponse rate which is 28. Therefore, the final sample size is 303.

### 2.7. Sampling Technique and Sampling Procedures

Simple random sampling technique was used to select the study subjects.

First, the list of patients was obtained from the database and registration logbook. Then, the lottery method was used to select each study subject.

### 2.8. Data Collection Instrument and Procedure

Data was collected using pretested interviewer-administered questionnaire, patient card review. Generally, questionnaire for this study contains 57 items and takes 30-45 minutes.

*Part I*. *Socioeconomic characteristics* were developed based on reviewed literature [8]. This contains 10 items with both open-ended and close-ended questions.

*Part II*. *Behavioral factors* are questions pertaining to substance use and sexual practices developed based on revied literature [41]. This contains 05 items with close-ended questions.

*Part III*. Clinical factors are from some of the questionnaires adapted from another literature [36]. This contains 10 items with both open- and close-ended questions and Morisky Medication Adherence Scale. Eight questionnaires were standard which contains 08 items with close-ended questions; adherence was defined as adherent (high) with a Morisky Medication Adherence Scale score of 0 and nonadherent (medium, low) with a score of 1 [44, 45]. The reliability of the instrument calculated using Cornbrash's alpha coefficient was 0.725.

*Part IV*. *Patient Health Questionnaire (PHQ-9) depressive symptom scale* contains 09 items. It was a Likert scale with the following response options:Pateint health questionnaire (PHQ-9) depressive symptom scale have 09 items. 0 = not at all, 1 = several days, 2 = more than half the days, and 3 = nearly every day. Classification of depression was measured by verbal responses of participants to PHQ-9 scale and expressed in scores. PHQ-9 was categorized as follows: severe depression: respondent with a score between 20 and 27; moderately severe depression: respondent with a score between 15 and 19; moderate depression: respondent with a score between 10 and 14; mild depression: respondent with a score between 5 and 9; minimal depression: respondent with a score between 1 and 4; and nondepressed respondent with score 0 [46, 47]. The internal consistency of this instrument using Cornbrash's alpha was 0.883.

*Part V*. *Psychosocial factors: Oslo Social Support Scale* contains 03 items by using close-ended questions. It was standard measured by verbal responses of participants to Oslo Social Support Scale and expressed in scores. Social support is categorized as follows: strong social support: respondent with a score between 12 and 14; moderate social support: respondent with a score between 9 and 11; and poor social support: respondent with a score between 3 and 8 [48]. The instrument showed reliability value 0.721, by Cornbrash's alpha.

*Perceived HIV related stigma* contains 12-item standard Likert scale with the following response options: 1 = strongly disagree, 2 = disagree, 3 = agree, and 4 = strongly agree. It contains personalized stigma, disclosure, public attitudes, and negative self-image. Perceived HIV-related stigma is assessed with a Likert scale[48]. The Cornbrash's alpha coefficient value was 0.648.

### 2.9. Data Processing and Analyses

The data were analyzed by SPSS version 23, using simple descriptive statistics.

The presence of crude association between the dependent variable with each independent variable was determined by bivariate logistic regression analysis. Variables that showed statistically significant association by bivariate analysis (*p* < 0.25) were further subjected to multivariable logistic regression analysis to control the confounding variables. The significance of association was determined at 95% confidence interval and *p* value < 0.05.

### 2.10. Data Quality Management

Two days of training were given to data collectors (four BSc nurses) and supervisors (two BSc nurses) on the objectives, clarity of tools, and overall data collection procedures to standardize interview procedures and reduce interviewers' bias. The final version of the questionnaire was translated into the Afan Oromo and Amharic language and once again retranslated in English language by bilingual experts, to check for language consistency of the tool.

The questionnaires were pretested on 5% of the sample at Shenen Gibe Hospital to determine clarity of the tool and feasibility of the study. At the end of each day, during data collection period, the collected data were checked for consistence and completeness.

### 2.11. Ethical Consideration

The study was conducted after the investigator gets an approval letter from the Institutional Review Board of Jimma University. Permission letter was provided to the hospital administrator before data collection. The purpose and procedure of data collection were clearly stated for the participants, and confidentiality and privacy were ensured. The right to refuse or withdraw from the study was respected. There were no risks or hazards to the participants, and there was no incentive for the participants. Those participants found to have suicide attempt and severe depression were referred to the psychotic clinics for early treatment.

## 3. Results

### 3.1. Socioeconomic Characteristics of the Participants

All the 303 patients who were selected for the study voluntarily responded to the interview, giving 100% response rate. More than half of the respondents, 159 (52.5%), were females. Maximum representation was by patients who were in the productive age group 25-44 yrs, 202 (66.7%) with mean age 39.36 (SD ± 9.63 years); they were married (125 (41.3%)) and lived with their families (217 (71.6%)). Most had primary education (127 (41.9%)) and were employed (100 (33%)). Urban residents constituted the majority (269 (88.8%)), and 203 (67%) were earning less than 1380 ETB per month. They were of Oromo ethnicity (145 (47.9%)), Orthodox Christians (121 (39.9%)) with moderate social support (143 (47.2%)) (Table 1).

### 3.2. Prevalence of Depression

**Figure 1:**
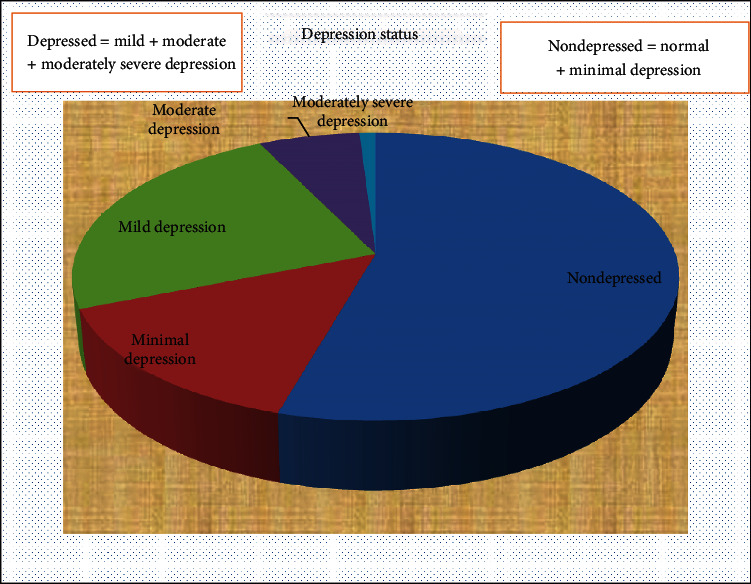
Prevalence of depression among study participants, Jimma University Medical Center, April to May 2018 (*N* = 303).

Nearly one-third (94 (31%)) of the participants were found to have depression, while 72 (24%) had mild depression, 19 (6%) had moderate depression, and 3 (1%) were found to be with moderately severe depression (Figure 1).

### 3.3. Substance Use and Risky Behavior

Interview on the behavioral characteristics revealed that few numbers of the respondents (53 (17.5%)) used alcohol, 7 (2.3%) of the participants smoked tobacco, and 78 (25.7%) of the participants chewed khat. Information on the sexual practices showed that 25 (67.6%) reported inconsistent use of condom whereas 12 (32.4%) never used a condom (Table 2).

### 3.4. Clinical Characteristics of the Participants

Most of the participants (290 (95.7%)) were at HIV stage T1, 125 (41.3%) had been diagnosed as HIV infected for the past 6-10 years, and 128 (42.2%) had been on treatment since the last 6-10 yrs.

While most participants (226 (74.6%)) had never been screened for depression prior to initiation of ART, 155 (51.2%) received treatment with 1e/Tdf-3tc-Nvp. Majority of the participants (264 (87.1%)) did not experience side effect from ART and 218 (71.9%) did not have opportunistic infection, 147 (48.5%) had recent CD4 cell count which was between 200 and 499 cells/mm^3^, and 228 (75.2%) of the participants had never received food supplement from ARV treatment programmer. But more than half of the respondents (164 (54.1%)) reported good adherence to ART (Table 3).

### 3.5. Aspects of Stigma

The HIV/AIDS-related stigma scale showed personal stigma mean score 6.23 SD ± 1.572, disclosure mean score 7.49 SD ± 1.819, public attitude mean score 5.78 SD ± 1.060, and self-image mean score 5.88 SD ± 1.386 (Table 4).

### 3.6. Factors Associated with Depression among People Living with HIV/AIDS

Among all the explanatory variables subjected to bivariate binary logistic regression analysis, sex of participant, marital status, occupation, social support, medication adherence, alcohol drinking, khat chewing, opportunistic infection, and CD4 count had *p* value < 0.25 and were further entered into multivariable analysis to control for confounding.

In multivariable logistic regression, four independent predictor variables (sex, marital status, opportunistic infection, and medication adherence) were found to be significantly associated with depression at *p* value less than 0.05.

Susceptibility for depression was more among HIV-infected females (AOR: 0.510, 95% CI: 0.264-0.986), widowed persons (AOR: 3.610, 95% CI: 1.649-7.901), contracting opportunistic infections (AOR: 3.122, 95% CI: 2.719-61.923), and poor medication adherence (AOR: 0.470, 95% CI: 0.266-0.831) (Table 5).

## 4. Discussion

This study was conducted to investigate the prevalence of depression among PLWHA enrolled in ART and its association with sociodemographic, behavioral, clinical, and social support factor.

### 4.1. The Prevalence of Depression

This study revealed that the prevalence of depression among the study participants was 94 (31%) and was lower than that of the studies conducted in Ethiopia at Debre Birhan (38.94%) and in northern Showa (76.7%) [41, 8]. Studies done in India showed that the prevalence of depression was 58.75% and 67.3% at different periods [7, 32] similarly in Brazil, Denmark, and north central Nigeria (42.3%, 61%, and 56.7%, respectively) [29, 30, 33], but higher than the prevalence among patients at Hawassa, Ethiopia (24.5%) [49].

The variations could be attributed to differences in sample size, instruments used to determine depression, and different geographical locations. Moreover, it is likely that responding by a face-to-face interview may have prompted the participants to give socially desirable answers. However, this very significant finding in the current study highlights the need for early diagnosis of depression in this vulnerable population.

### 4.2. Factors Associated with Depression

Research shows gender is closely associated with depression with women being more prone to depression. This study confirmed that HIV-infected males were 49% less likely to develop depression when compared to females. Similar findings were shown in studies from northern Showa, north central Nigeria, Tehran, and Hawassa [8, 33, 37, 49]. Women in male-dominated societies are at the receiving end for all the misfortunes such as poverty, violence, discrimination, and single parenthood. Being diagnosed with HIV adds to this burden and makes them more vulnerable to depression than males. The findings are negated by the study at Harar region of Ethiopia [36].

This study found that the marital status of the HIV-infected person is a significant factor for comorbidity with depression. Widowhood predisposed a person nearly 4 times to develop depression when compared to those who had a stable marital life. The findings are in line with studies conducted in Harar and Hawassa (Ethiopia) [36, 37] and Tehran in Iran [49]. Unstable marital relationships and loss of partner predispose for depression, but presence of good social support can be a buffer against stress and depression.

Another major finding of this study was the risk for depression increased threefold when the person contracts opportunistic infections, which is in line with a study at Fiche, Ethiopia [8]. Opportunistic infections have synergistic effects on the disease progression and AIDS-related mortalities. Depression has an effect on immunosuppression, and immunosuppression inversely predisposes depression.

Good medication adherence can lower the risk for depression by more than half as revealed through this study and supported by similar studies in Addis Ababa, Ethiopia [42]. HIV-infected people often default on medication due to difficulty in accessing the health center, being forgetful and due to unpleasant side effects of antiretroviral therapy. This significant finding reemphasizes the need for creating awareness on medication compliance among HIV-infected people and providing supportive services for individuals on treatment.

It is perplexing that this study did not find a significant association between depression and social support, perceived stigma, substance use, and CD4 count among the respondents, contrary to available literature. The recent CD4 cell count of most participants in this study was between 200 and 499 cells/mm^3^. Moreover, the tendency to give socially desirable responses in an interview may be a reason for this contradiction.

Individual's sex, marital status, opportunistic infections, and medication adherence were factors associated with risk of developing depression among people living with HIV/AIDS.

## Figures and Tables

**Table 1:** Distribution of study participants by their socioeconomic characteristics, Jimma University Medical Center, April to May 2018 (*N* = 303).

Characteristics	*N*	%
Sex		
Male	144	47.5
Female	159	52.5
Total	303	100
Age		
18-24	13	4.3
25-44	202	66.7
45-64	80	26.4
>65	8	2.6
Total	303	100
Marital status		
Married	125	41.3
Single	42	13.9
Divorced	74	24.4
Separated	6	2.0
Widowed	56	18.5
Total	303	100
Living status		
With family	217	71.6
Alone	53	17.5
With relatives	33	10.9
Total	303	100
Educational status		
No education	63	20.8
Primary school	127	41.9
Secondary school	42	13.8
Above secondary	71	23.4
Total	**303**	100
Occupation		
Farmer	18	5.9
Government employee	100	33.0
Student	12	4.0
Daily laborer	91	30.0
Housewife	68	22.4
Unemployed	14	4.6
Total	303	100
Residence		
Rural	34	11.2
Urban	269	88.8
Total	303	100
Monthly income		
<1380	203	67.0
1381-2872	45	14.9
>2872	55	18.2
Total	303	100
Ethnicity		
Oromo	145	47.9
Amhara	82	27.1
Tigre	19	6.3
Gurage	26	8.6
Dawuro	31	10.2
Total	303	100
Religion		
Muslim	105	34.7
Orthodox	121	39.9
Protestant	63	20.8
Catholic	14	4.6
Total	303	100
Social support		
Poor support	117	38.6
Moderate support	143	47.2
Good support	43	14.2
Total	303	100

**Table 2:** Distribution of study participants by their behavioral characteristics, Jimma University Medical Center, April-May 2018 (*N* = 303).

Characteristics	*N* = 303			
	Male	Female	Total	Percent
Alcohol use				
Yes	28	25	53	17.5
No	116	134	250	82.5
Tobacco use				
Yes	5	2	7	2.3
No	139	157	296	97.7
Khat use				
Yes	44	34	78	25.7
No	100	125	225	74.3
Unsafe sex practice				
Yes	22	15	37	12.2
No	122	114	266	87.8
Condom utilization				
Inconsistent condom use	15	10	25	67.6
Never use a condom	1	3	4	32.7

**Table 3:** Distribution of study participants by their clinical characteristics, Jimma University Medical Center, April-May 2018 (*N* = 303).

Characteristics	*N* (*n* = 303)	%
Side effects of ART		
None	264	87.1
Sometimes	29	9.6
Always	10	3.3
Diagnosed with OIs in the past 6 months		
None	218	71.9
Once and twice or more	85	28.1
Recent CD4 level		
<200	7	2.3
200-499	147	48.5
>500	149	49.2
Chronic disease		
Yes	80	26.4
No	223	73.6
Screened for depression prior to ART		
Yes	77	25.4
No	226	74.6
Stage of HIV/ADS		
T1	290	95.7
T2	13	4.3
Type of ART regimen		
1c	81	26.7
1d	30	9.9
1e	155	51.2
1f	28	9.2
2h	9	3.0
Duration on HAART treatment (in months)		
4-40	41	13.5
41-80	104	34.3
81-120	128	42.2
>120	30	9.9
Duration from diagnosis HIV infection (in months)		
4-40	37	12.2
41-80	103	34.0
81-120	125	41.3
>120	38	12.5
Receive food supplement from ARV treatment programmer		
Never	228	75.2
Previously	66	21.8
Currently	9	3.0
Medication adherence		
Good adherence	164	54.1
Poor adherence	139	45.9

**Table 4:** Distributions of study participants by their stigma characteristics, Jimma University Medical Center, April-May 2018 (*N* = 303).

Characteristics	Mean	Standard deviation	Item
Personal stigma	6.23	1.572	3
Disclosure	7.49	1.819	3
Public attitude	5.78	1.060	3
Self-image	5.88	1.386	3

**Table 5:** Bivariate binary logistic regression analysis and multivariable logistic regression analysis of the respondents in JUMC, April to May 2018.

Characteristics	Depression		COR (95% CI) *p* value	AOR (95% CI) *p* value
	None	Depressed		
Sex				
Male	107	37	0.619 (0.377-1.015)∗	0.510 (0.264-0.986)∗∗
Female	102	57	1	1
Marital status				
Married	97	28	1	1
Single	32	10	0.310 (0.158-0.607)∗	0.889 (0.318-2.486)
Divorced	46	28	0.336 (0.139-0.811)∗	1.638 (0.752-3.568)
Separated	97	28	0.654 (0.323-1.322)∗	0.778 (0.079-7.635)
Widowed	32	10	0.215 (0.024-1.958)∗	3.610 (1.649-7.901)∗∗
Occupation				
Farmer	12	6	1	1
G. employee	74	26	1.833 (0.367-9.166)	2.680 (0.443-16.220)
Student	9	3	1.288 (0.333-4.982)	2.418 (0.517-11.303)
Daily laborer	53	38	1.222 (0.197-7.594)	1.395 (0.147-13.226)
H. wife	50	18	2.629 (0.686-10.069)∗	3.757 (0.800-17.642)
Unemployed	11	3	1.320 (0.330-5.276)	1.232 (0.246-6.178)
Alcohol use				
Yes	31	22	1.754 (0.952-3.233)∗	1.590 (0.715-3.537)
No	178	72	1	1
Khat use				
Yes	49	29	1.457 (0.847-2.505)∗	1.482 (0.717-3.063)
No	160	65	1	1
Diagnosed with OIs				
None	166	52	1	1
Once and twice or more	43	42	0.321 (0.189-0.543)∗	3.122 (1.700-5.733)∗∗
CD4 level				
<200	5	2	1	1
200-499	93	54	1.168 (0.218-6.274)	0.860 (0.119-6.192)
>500	111	38	1.696 (1.031-2.791)	1.096 (0.602-1.997)
Medication adherence				
Good adherence	105	59	2.343 (1.475-3.723)∗	0.470 (0.266-0.831)∗∗
Poor adherence	60	79	1	1
Social support				
Poor support	66	51	1	1
Moderate support	110	33	2.550 (1.150-5.654)∗	1.956 (0.785-4.871)
Good support	33	10	0.990 (0.442-2.220)	1.074 (0.428-2.697)

NB: ∗*p* < 0.25, ∗∗*p* < 0.05, 1 = reference.

## Data Availability

The data used to support the findings of this study are included within the article.

## Abbreviations

AIDS: Acquired immune deficiency syndrome
AOR: Adjusted odds ratio
ART: Antiretroviral therapy
BDI: Beck Depression Inventory
BMI: Body mass index
CI: Confidence interval
COR: Crude odds ratio
ETB: Ethiopian birr
JUMC: Jimma University Medical Center
MMAS: Morisky Medication Adherence Scale
OIs: Opportunistic infections
OR: Odds ratio
PHQ: Patient Health Questionnaires
PLWHA: People living with HIV/AIDS
SPSS: Statistical Package Software for Social Sciences
WHO: World Health Organization.
